# The human SIN3B corepressor forms a nucleolar complex with leukemia-associated ETO homologues

**DOI:** 10.1186/1471-2199-9-8

**Published:** 2008-01-19

**Authors:** Rakesh Singh Dhanda, Sofia Rondin Lindberg, Inge Olsson

**Affiliations:** 1 Department of Hematology, C14, BMC, SE-221 84 Lund, Sweden

## Abstract

**Background:**

SIN3 (SWI-Independent) is part of a transcriptional deacetylase complex, which generally mediates the formation of repressive chromatin. The purpose of this work was to study possible interactions between corepressors human SIN3B (hSIN3B) and the ETO homologues – ETO (eight twenty-one), MTG16 (myeloid-transforming gene 16) and MTGR1 (MTG-related protein 1). In addition, the subnuclear localization of the hSIN3B and the ETO homologues was also examined.

**Results:**

A ubiquitous expression of hSIN3B was observed in adult and fetal tissues. Results with both ectopically expressed proteins in COS-7 cells and endogeneous proteins in the K562 human erytholeukemia cell line demonstrated interactions between hSIN3B and ETO or MTG16 but not MTGR1. Furthermore, nuclear extract of primary placental cells showed complexes between hSIN3B and ETO. The interaction between hSIN3B and ETO required an intact amino-terminus of ETO and the NHR2 domain. A nucleolar localization of hSIN3B and all the ETO homologues was demonstrated upon overexpression in COS-7 cells, and confirmed for the endogeneously expressed proteins in K562 cells. However, hSIN3B did not colocalize or interact with the leukemia-associated AML1 -ETO.

**Conclusion:**

Our data from protein-protein interactions and immunolocalization experiments support that hSIN3B is a potential member of a corepressor complex involving selective ETO homologues.

## Background

Genes are regulated by sequence-specific DNA-binding transcription factors and interacting partner proteins [1]. These physically interacting partners form complexes that are responsible for chromatin modifications. The SWI-Independent (SIN3) [2] (also called SDI1) [3] corepressor serves as an essential scaffold for several proteins. By recruitment of histone deacetylases (HDACs), it forms a deacetylase complex which generally catalyzes the silencing of the promoter [4-7]. SIN3 can either interact directly with transcription factors or indirectly through adapter molecules like NCoR (the nuclear receptor corepressor) and/or SMRT (silencing mediator of retinoid and thyroid hormone receptor). The SIN3 homologues have four evolutionarily conserved paired amphipathic helix (PAH) regions, a histone deacetylase interaction domain (HID) and a highly conserved region (HCR) [8]. An increasing number of nuclear proteins have been observed to interact with SIN3 in a flexible manner. As a result, SIN3 can both suppress and activate gene promoters [9].

The SIN3 family consists of SIN3A and SIN3B, which are highly homologous. Human SIN3B (hSIN3B) contains 1,130 amino acids, which share 90% identity with murine SIN3B (mSIN3B) [10]. Interactions between murine SIN3A (mSIN3A) and corepressor proteins such as the ETO homologues have been reported [11-13]. The ETO homologues include the transcriptional corepressors ETO (or MTG8), MTG16 and MTGR1 [14-17]. mSIN3A has been shown to associate with ETO [11-13], but not with ETO-2, the murine homologue of MTG16 [11]. Endogenously, ETO is known to associate with mSIN3A, N-CoR and HDACs [12,13]. However, interaction studies between hSIN3B and the ETO homologues have not yet been reported.

The ETO homologues are evolutionarily related to the Drosophila protein Nervy, sharing four conserved regions: Nervy homology regions (NHR) 1–4 [18]. NHR2 and the flanking regions to NHR2 are required for the interaction of ETO with mSIN3A [19,20]. The ETO homologues do not bind directly to DNA but rather repress transcription indirectly by binding to nuclear corepressor proteins such as NCoR, SMRT and mSIN3A [11-13,21,22]. Obviously, mSIN3A is part of a corepressor complex that can include ETO as one element. In the present work, we investigated whether hSIN3B can also bind to the ETO homologues.

Both ETO and MTG16 are known to carry out transcriptional repression as components of chimeric proteins generated by chromosomal translocations in certain subtypes of acute myeloid leukemia (AML). The t(8;21) gives rise to the AML1-ETO fusion gene [23,24], and the t(16;21) gives rise to the AML1-MTG16 fusion gene [14]. The leukemia fusion proteins can recruit corepressors and HDACs, leading to dysregulated transcriptional repression that is responsible for a block in cell differentiation [25]. AML1-ETO retains the DNA-binding region of AML1, but the transactivation domain is deleted. However, the ETO partner of AML1-ETO retains the conserved regions NHR1-4, allowing interactions with corepressors. AML1-ETO has been shown to interact with mSIN3A [12,13].

Additional studies of the interactions between different isoforms of SIN3 and their partners in the transcriptional deacetylase complex may provide new knowledge about gene regulation. Therefore, in the current study we examined the interactions of hSIN3B with the ETO homologues as well as with AML1-ETO. Our results demonstrate formation of complexes between hSIN3B and selective ETO homologues both upon ectopic coexpression in COS-7 cells and, more importantly, endogeneously in primary placenta cells and the K562 human erythroleukemia cell line. Furthermore, immunolocalization studies showed that hSIN3B and ETO homologues colocalized to the nucleolus. Our results suggest that hSIN3B is a member of a corepressor complex involving specific ETO homologues.

## Results

### Tissue and cell line expression of hSIN3B and the ETO homologues

**Figure 1:**
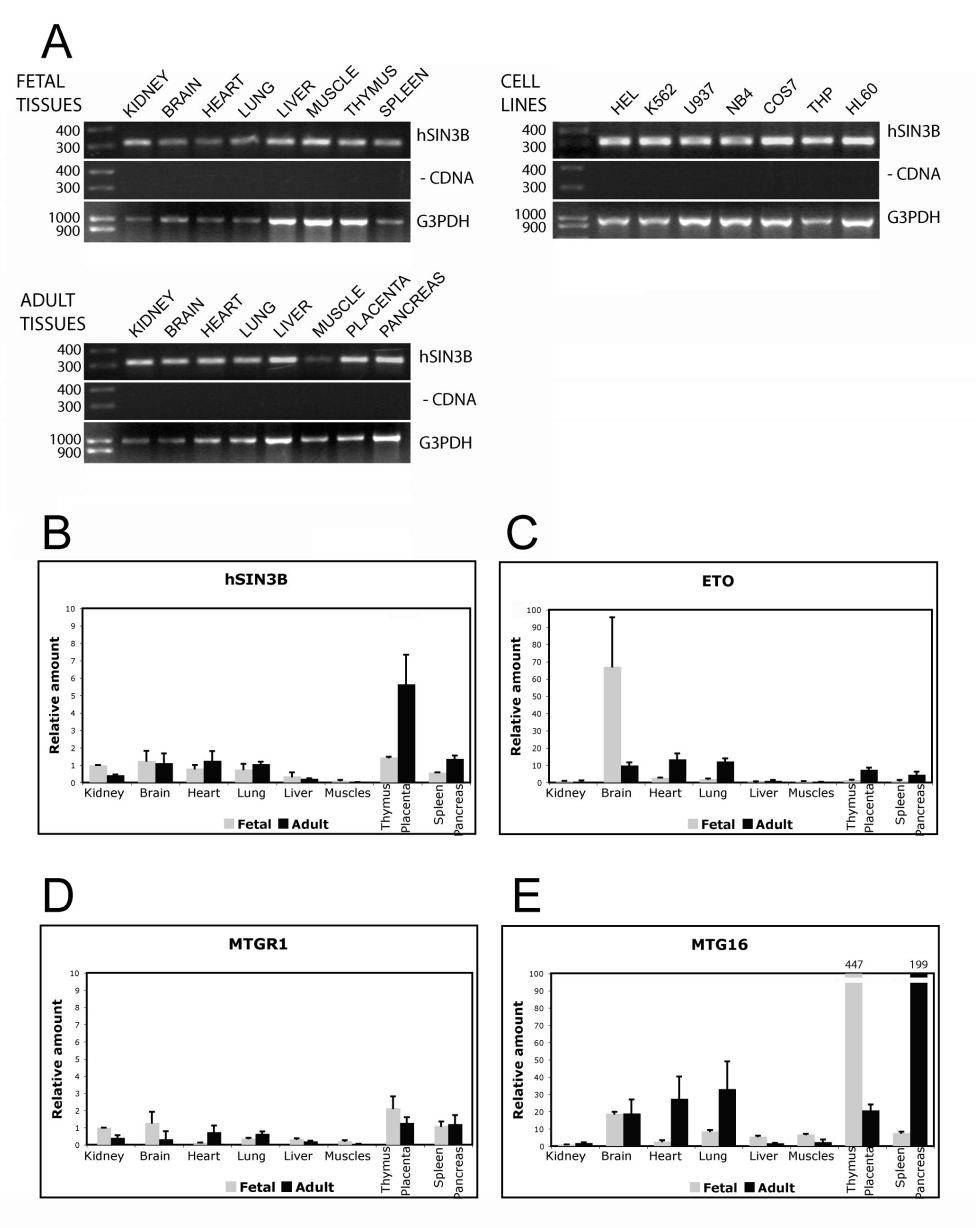
**Tissue expression of hSIN3B and the ETO homologues**. (**A**) Expression of hSIN3B in various tissues and cell lines as measured by RT-PCR (Upper panels). Middle panels represent negative controls, wherein RT-PCR was carried out without cDNA. Glyceraldehyde-3-phosphate dehydrogenase (G3PDH) was used as an endogenous control (Lower panels). (**B-E**) Tissue expression of hSIN3B and ETO homologues as measured by real-time PCR. Relative transcript levels were calculated after normalizing with the G3PDH (see materials and methods). Three independent experiments, each in triplicate were performed and error bars show the Standard Deviation (S.D.).

First, an attempt was made to determine the general expression of hSIN3B by investigating several tissues. Results from RT-PCR showed hSIN3B mRNA to be expressed in all tissues and cell lines examined (Fig. 1A). Because the transcript levels reach saturation during RT-PCR, the results may not reflect the true number of transcripts. Therefore, we also carried out real-time PCR, which showed that hSIN3B and ETO homologues are ubiquitously expressed with a variable number of transcripts (Fig. 1B–E). The highest expression of hSIN3B was found in thymus, placenta, pancreas, brain, heart and lung. In general, these tissues also had the highest expression of the ETO homologues confirming earlier results [17]. Furthermore, liver and muscle showed the lowest transcript levels for both hSIN3B and the ETO homologues. The other tissues showed no covariation between hSIN3B and ETO homologues.

### hSIN3B interacts with selective ETO homologues

**Figure 2:**
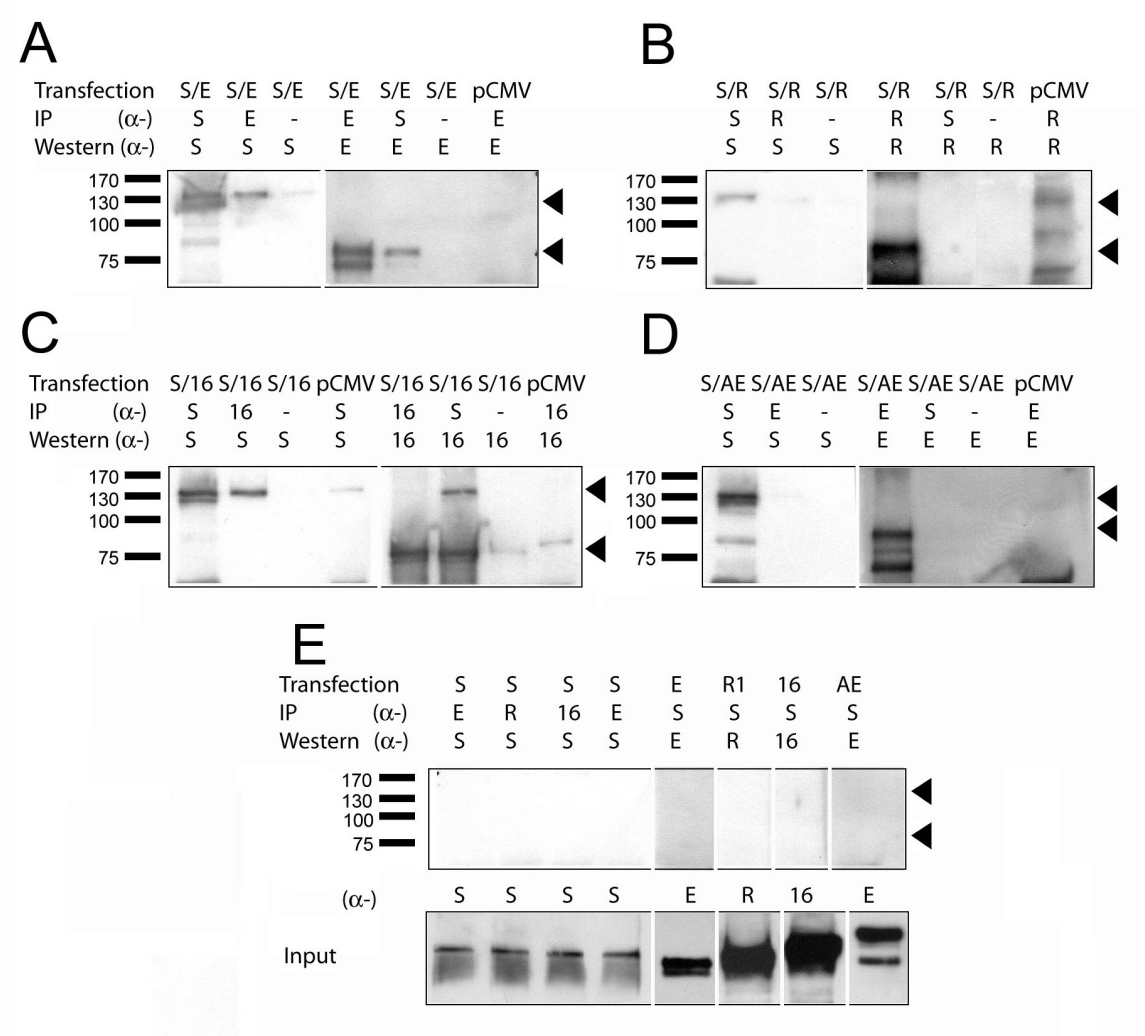
**hSIN3B interacts with ETO homologues, but not with AML1-ETO**. COS-7 cells were transfected with ETO (E), MTGR1 (R), MTG16 (16) or AML1-ETO (AE) in combination with hSIN3B (S). Cell lysates were analyzed by IP-Western, as described in Materials and Methods. IP and Western were performed with α-E (ETO specific), α-R (MTGR1 specific), α-16 (MTG16 specific) and α-S (hSIN3B specific). (**A-C**) ETO and MTG16 co-precipitated hSIN3B (lane 2 of A and C). Reciprocal experiments showed that hSIN3B co-precipitated ETO and MTG16 (lane 5 of A; lane 6 of C). MTGR1 did not co-precipitate hSIN3B or vice-a-versa (lanes 2 and 5 of B). Arrowheads show the position of hSIN3B corresponding to approximately 135 kDa and ETO corresponding to approximately 75 kDa (lanes 2 and 4). (**D**) AML1-ETO failed to co-precipitate hSIN3B (lane 2). The reciprocal experiment with the same lysates showed that hSIN3B did not precipitate AML1-ETO (lane 5). The size of AML1-ETO is about 100 kDa. **(E) **Control experiments showed that none of the antibodies bound unspecifically. Lower panel shows the input of hSIN3B and ETO homologue in 2% of IP lysate. The positions of the molecular weight markers are indicated at the left.

ETO has previously been shown to be present in an endogeneous complex containing mSIN3A [12]. SIN3 proteins are large and thus suitable for cooperation with multiple nuclear partners [9]. For these reasons, we investigated possible interactions between hSIN3B and ETO homologues. To determine this, transient transfections were carried out in COS-7 cells followed by IP-Western analyses. Control experiments showed that none of the antibodies used in these experiments (α-ETO, α-MTGR1, α-MTG16 and α-SIN3B) bound non-specifically (Fig. 2E). Three independent experiments were performed and typical data is shown in Fig. 2. IP was performed with α-ETO and Western blotting with α-hSIN3B on extracts from cells co-expressing hSIN3B and ETO. As a result, a protein of approximately 135 kDa was pulled down, corresponding to the size of hSIN3B (Fig. 2A, lane 2). Thus, ETO co-precipitated hSIN3B. The reciprocal IP-Western experiment demonstrated that hSIN3B co-precipitated ETO as a 75 kDa protein (Fig. 2A, lane 5) further strengthening the conclusion that hSIN3B and ETO can form a complex. As hSIN3B interacted with ETO, we also investigated whether hSIN3B can interact with the chimeric oncoprotein AML1-ETO. However, hSIN3B was not co-precipitated by AML1-ETO and in the reverse experiment AML1-ETO was not co-precipitated by hSIN3B (Fig. 2D, lanes 2 and 5) indicating a lack of interaction. An interaction was also shown between hSIN3B and MTG16 (Fig. 2C), but not between hSIN3B and the ETO homologue MTGR1 (Fig. 2B).

**Figure 3:**
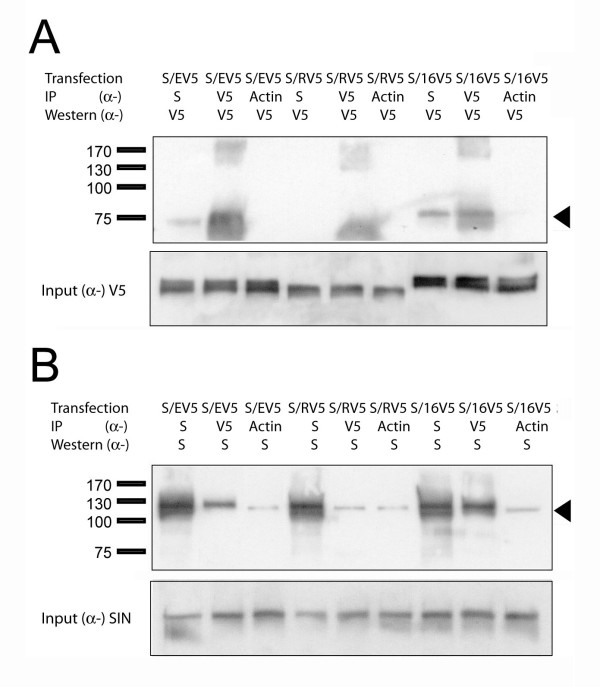
**hSIN3B interacts with V5-tagged ETO homologues**. COS-7 cells were transfected with pCMV_ETO-V5 (EV5) pCMV_ MTGR1-V5 (ER5), pCMV_ MTG16 -V5 (16V5) in combination with hSIN3B (S) as described in Materials and Methods. IP and Western were performed with α-V5 (V5 specific) and α-S (hSIN3B specific). (**A-B**) ETO and MTG16 co-precipitated hSIN3B (Lanes 1 and 7 of A respectively). Reciprocal experiments showed that hSIN3B co-precipitated ETO (Lanes 2 and 8 of B respectively). Arrowhead shows the position of ETO in A and MTGR1 in B. MTGR1 failed to co-precipitate hSIN3B (Lane 4 of A). The reciprocal experiment showed that hSIN3B did not precipitate MTGR1 (Lane 5 of B). Lower panels in A and B show input of hSIN3B and ETO homologue in 2% of IP lysate. The positions of the molecular weight markers are indicated at the left.

In order to confirm the specificity of these interactions experiments were also carried out using ETO homologue constructs tagged with V5 and detected by anti-V5. Three independent experiments were performed by expression in COS-7 cells and typical data is shown in Fig. 3. hSIN3B pulled down ETO-V5 and MTG16-V5 but not MTGR1-V5 (Fig. 3A). In the reverse experiment ETO-V5 and MTG16-V5 but not MTGR1-V5 pulled hSIN3B (Fig. 3B), Thus, the results confirmed the specificity of interaction between SIN3B and ETO homologues; SIN3B interacted with ETO and MTG16 but not with MTGR1. From the present results, we conclude that hSIN3B can form stable interactions with selective ETO homologues, but not with AML1-ETO.

We also tried to confirm the interactions between hSIN3B and ETO homologues in a mammalian two-hybrid assay. However, a repressor activity of the ETO homologue constructs might have lowered the signals making it difficult to differentiate between interaction of the molecules in this system (data not shown).

### The ETO domain NHR2 and the amino-terminus are required for interaction with hSIN3B

**Figure 4:**
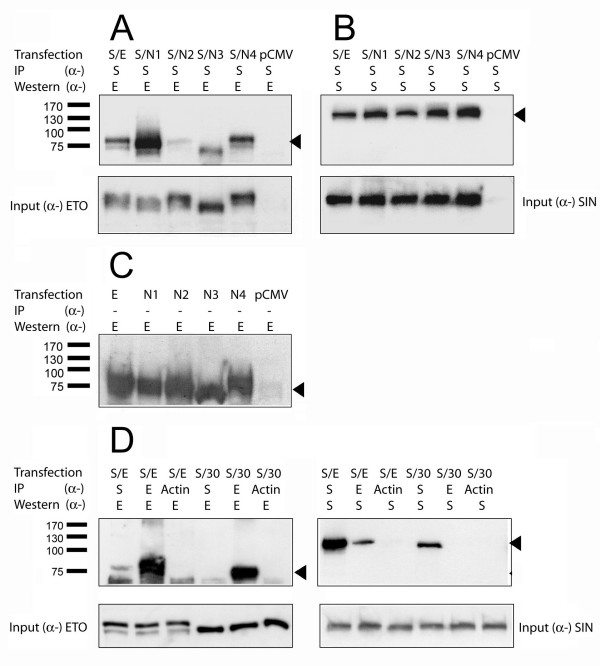
**NHR2 and amino terminal domain of ETO are involved in interaction with hSIN3B**. Lysates were prepared from COS-7 cells transfected with hSIN3B (S) in combination with ETO (E) and ETO mutants lacking NHR1 (N1), NHR2 (N2), NHR3 (N3), NHR4 (N4) or ETO lacking 30 amino-terminal aminoacids (30) as described in Material and Methods. **(A) **IP was performed with α-SIN3B followed by Western blotting with α-ETO (α-E). The ETO mutant lacking NHR2 was not pulled down by hSIN3B (lane 3). **(B) **Both IP and Western blotting with α-SIN3B (α-S) confirmed expression of hSIN3B (arrowhead). **(C) **Western blotting performed with α-E demonstrated expression of all the mutants (arrowhead). **(D) **IP was performed with α-SIN3B or α-ETO followed by Western blotting. The presence in the left panel of ETO in lane 1 but not in lane 4 (lacking 30 amino-terminal aminoacids) showed that the ETO amino-terminus is required for interaction with hSIN3B. Reciprocal experiment showed that hSIN3B co-precipitated ETO (right panel, lane 2) but not ETO – 30 aminoacids (right panel, lane 5). Arrowheads show the position of ETO, ETO – 30 aas and hSIN3B. Lower left and right panels show input of ETO and hSIN3B in 2% of IP lysate. The positions of the molecular weight markers are indicated at the left.

Of the four evolutionary conserved regions (NHRs) of ETO homologues, a region spanning from NHR2 to NHR4 has been described to associate with corepressors such as SIN3A, N-CoR and SMRT [11-13,19,20]. Similarly, we wanted to identify the regions of ETO involved in the interaction with hSIN3B. For this we used ETO mutants lacking individual NHRs in cotransfection experiments in COS-7 cells. Three independent experiments were performed and typical results are shown in Fig. 4. Immunoprecipitation was performed with α-SIN3B followed by Western blotting with α-ETO. Deletion of NHR2 abrogated co-precipitation of the ETO mutants by hSIN3B (Fig. 4A, lane 3). In contrast, deletion of NHR1, NHR3 or NHR4 did not interfere with the formation of complexes with hSIN3B (Fig. 4A, lanes 2, 4 and 5). The expression of hSIN3B was confirmed by performing IP-Western with α-hSIN3B (Fig. 4B, lanes 2–5), while the expression of ETO mutants was confirmed by Western blotting with α-ETO (Fig. 4C).

The ETO part of the leukemia fusion protein AML1-ETO lacks 30 N-terminal aminoacids. Therefore, it was important to determine whether these are important for the interaction with hSIN3B. Deletion of these amino acids from the amino terminal region of ETO abrogated co-precipitation by hSIN3B (Fig. 4D, left panel, lane 4). The reciprocal experiment showed that this mutant did not co-precipitate hSIN3B (Fig. 4D, right panel, lane 5). Full length ETO was used a control to show normal interaction between hSIN3B and ETO (Fig. 4D, right panel, lane 1 and Fig. 4D, left panel, lane 2). Importantly, as evident from our previous result (Fig. 2D), AML1-ETO was unable to bind to hSIN3B. Our data indicate that both the amino-terminal part and NHR2 of ETO are required for the interaction with hSIN3B.

### Endogeneous hSIN3B co-immunoprecipitates ETO

**Figure 5:**
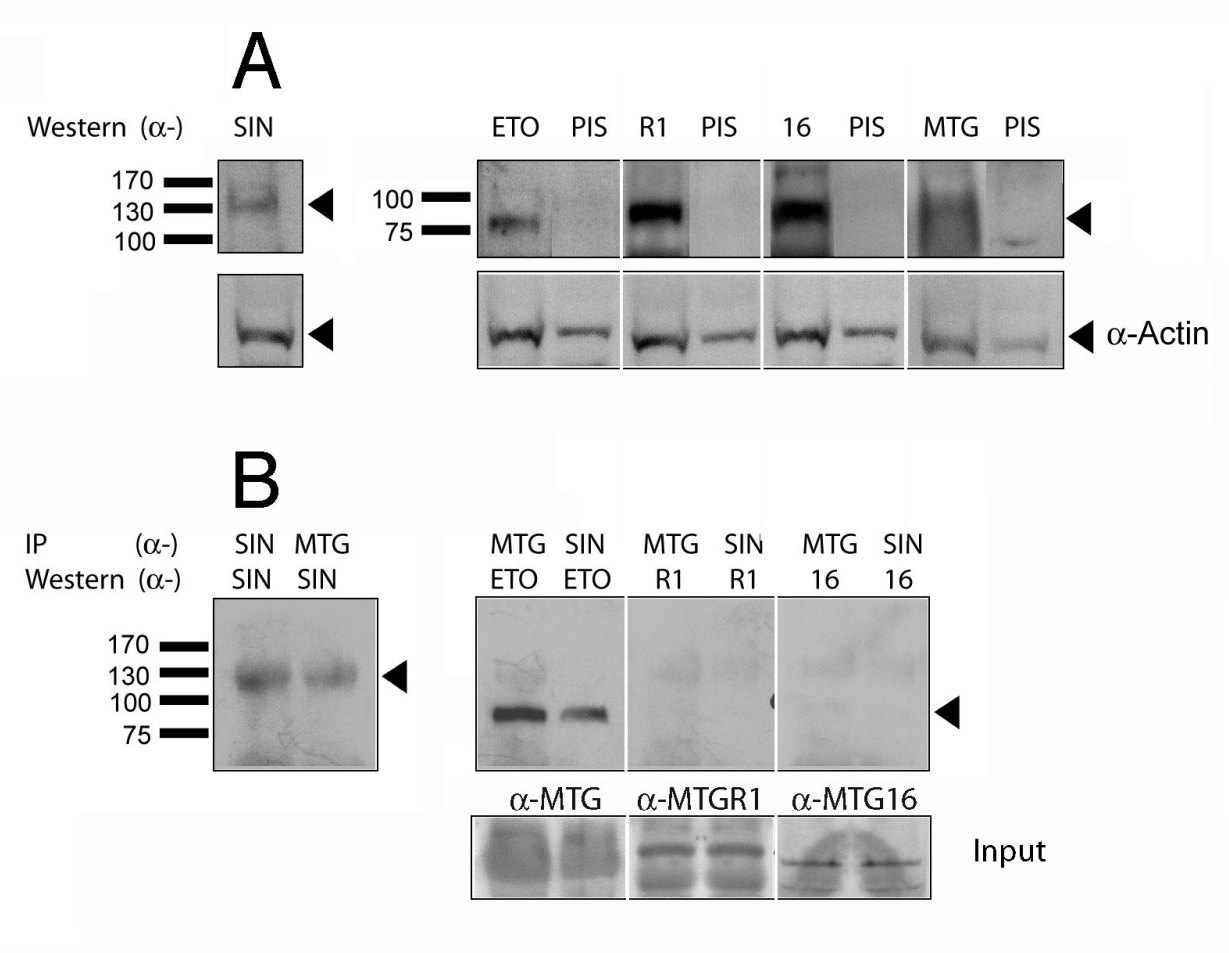
**hSIN3B interacts with ETO in primary placenta cells**. **(A) **Western blotting was used to detect hSIN3B and ETO homologues in TRIZOL extract of primary placental cells as described in Material and Methods. The following antibodies were used: α-hSIN3B (SIN), α-ETO (ETO), α-MTGR1 (R1), α-MTG16 (16) and α-MTG (MTG, reactive with all ETO homologues). Arrowhead shows the position of hSIN3B and ETO homologues. The blots were re-probed with pre-immune serum (PIS) in order to rule out non-specific binding of the antibodies. The blots were probed with α-actin to show equal loading. **(B) **IP-Western was used to examine the presence of complexes between hSIN3B and the ETO homologues in nuclear extracts. IP with α-MTG pulled down hSIN3B as detected on immunoblotting with α-SIN3B (SIN) (lane 2). In the reverse experiment, IP with α-SIN3B (SIN) pulled down ETO as detected on immunoblotting with α-ETO (ETO) (lane 4). However, IP with α-SIN3B (SIN) did not pull down MTGR1 or MTG16 as no signal was detected upon immunoblotting with α-MTGR1 or MTG16 (lanes 5–8). Lower panel shows input of MTGR1 and MTG16 in 2% of IP lysate. The positions of the molecular weight markers are indicated at the left.

The previous conclusions on interactions between hSIN3B and ETO homologues are based on data from overexpression in COS-7 cells (Fig. 2 and 3). Therefore, it was important to confirm the interactions between endogeneous proteins. For this purpose cells from the central villous part of the placenta were isolated. Results from Western blotting showed that hSIN3B and all the ETO homologues are present in the placental cells (Fig. 5A). To investigate whether ETO homologues were present in hSIN3B associated nuclear complexes, we immunoprecipitated nuclear placental cell extracts with α-SIN3B and performed Western blotting using ETO homologue specific antibodies. The reverse experiment was also carried out. The results show that ETO pulled down a protein of approximately 135 kDa, corresponding to hSIN3B (Fig. 5B, lane 2), and in the reverse experiment hSIN3B pulled down a protein of approximately 75 kDa, corresponding to ETO (Fig. 5B, lane 4). However, no co-immunoprecipitation was observed between hSIN3B and MTGR1 or MTG16 (Fig. 5B, lanes 6 and 8) though input data confirm the presence of MTGR1 and MTG16 in IP lysates. We were not able to show input of hSIN3B protein because of a low protein level in the lysate. Our data show that hSIN3B can interact with ETO in primary placental cells.

### Immunolocalization and antibody specificity

**Figure 6:**
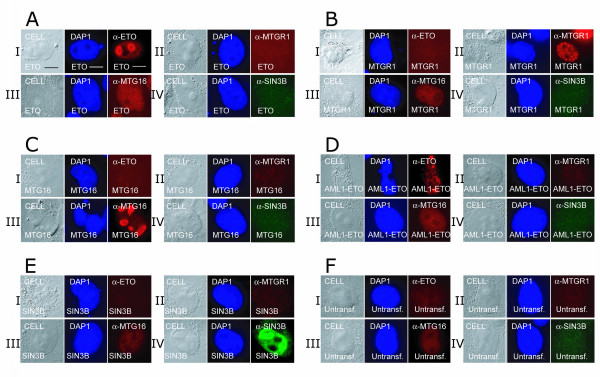
**Nuclear localization and antibody specificity**. **(A-E) **COS-7 cells were transfected with ETO, MTGR1, MTG16, AML1-ETO or hSIN3B (A to E respectively). Cells were immobilized, lysed and incubated with α-ETO, α-MTGR1, α-MTG16 or α-SIN3B as described in Materials and Methods. ETO (A I), MTGR1 (B II), MTG16 (C III), AML1-ETO (D I) and hSIN3B (E IV) were localized in the nucleus. **(F) **Untransfected (Untransf.) cells showed no immunofluroscence (I, II and IV) except for a faint background with α-MTG16 (III). Size bars (10 μM) are shown in panel AI.

The specificity of the peptide antibodies used against the ETO homologues in immunoprecipitation and Western blotting has been shown previously [26]. Furthermore, in immunolocalization assays using COS-7 cells, antibodies for hSIN3B (α-SIN3B) and ETO homologues (α-ETO, α-MTGR1 and α-MTG16) were also shown to bind specifically to their respective antigens (Fig. 6A, I; 5B, II; 5C, III; 5D, I; and 5E, IV). α-MTG16 showed a faint background in non-transfected cells, probably due to a low endogenous expression of MTG16 in these cells (Fig. 6A–E, III). However, this antibody produced a strong fluorescence signal in cells overexpressing MTG16 (Fig. 6C, III). A quantitative analysis of the DAPI stained cells showed the transfection efficiency for hSIN3B and ETO homologues to be 70–80% (Table 1).

**Table 1:** Transfection efficiency (%) of hSIN3B and ETO homologues.

Transfection	Cells-analyzed	α-SIN3B (%)	α-ETO (%)	α-MTGR1 (%)	α-MTG16 (%)
hSIN3B	149	68	0	0	0
ETO	128	0	83	0	0
MTGR1	143	0	0	68	0
MTG16	138	0	0	0	74
AML1-ETO	143	0	68	0	0
Untransfected	100	0	0	0	0

A total of 100 to 150 DAPI stained COS-7 cells successfully transfected with hSIN3B, ETO homologues or AML1-ETO were analyzed after probing with the respective antibodies. Untransfected cells did not show antibody binding (last row).

Our data confirm that the antibodies to hSIN3B and the ETO homologues specifically detect their respective antigens in immunofluorescence studies.

### Nucleolar localization of hSIN3B and ETO homologues

**Figure 7:**
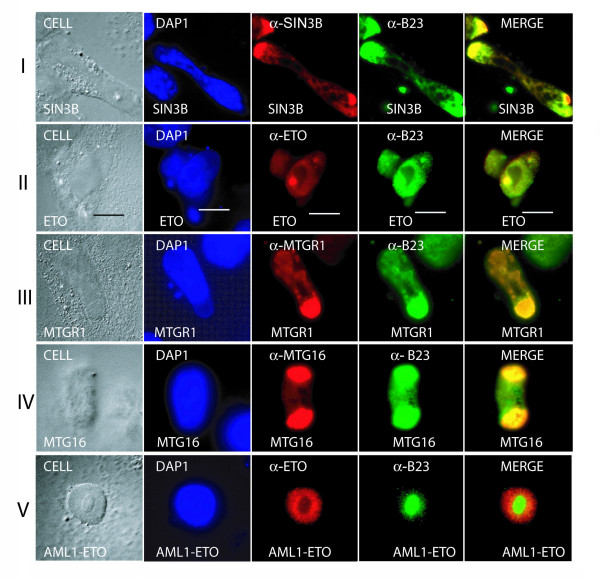
**Nucleolar localization of hSIN3B and ETO homologues**. The first column of each panel shows the Nomarski index. The second column shows nuclear staining with DAPI. The third column shows the localization of individual proteins. The fourth column shows nucleolar localization of the B23 nucleolar marker. The fifth column shows a merge of B23 and individual proteins. hSIN3B (I) and all the ETO homologues (II – IV) show colocalization with B23. AML1-ETO lacks colocalization with B23 (V). Size bars measuring 10 μM are shown in panel II.

A punctuate presence of all the ETO homologues in nuclear particles has been reported [13,27-29], and the presence of MTG16 in the nucleolus has also been reported [30]. Nuclear particles are formed at the end of the cell cycle [31,32] and are thought to migrate towards the nucleolar organizer region to fuse and aggregate into nuclear bodies, which eventually form nucleoli. B23 was used as a marker for nucleolar localization in COS-7 cells. Three independent experiments were performed and typical data are shown in Fig. 7. Cells expressing hSIN3B showed a nucleolar colocalization with B23 (Fig. 7, I). Moreover, all the ETO homologue transfected individually showed colocalization with B23 (Fig. 7, II – IV). However, AML1-ETO was not found in nucleoli but only in nuclear particles (Fig. 7, V).

A quantitative analysis of the data (Table 2) showed that 80–90 % of successfully transfected cells displayed an antibody signal in the nucleus only. Nucleoli (B23-positive) were observed in 11–20% of successfully transfected cells (% colocalization with B23 plus % no colocalization with B23 in Table 2). Approximately half of all nucleoli observed showed a signal for both hSIN3B and the individual ETO homologues judging by colocalization with B23. Thus, our data demonstrate that hSIN3B and the ETO homologues, but not AML1-ETO, can be targeted to the nucleolus.

**Table 2:** Quantitative analysis of nuclear and nucleolar localization of hSIN3B and ETO homologues.

Transfection	Cells analyzed	Nuclear signal only (%)	Nucleolar Signal with B23 (%)	Nucleolar signal without B23 (%)
hSIN3B	110	80	10	10
ETO	107	82	7	11
MTGR1	106	84	6	10
MTG16	113	84	12	4
AML1-ETO	415	89	0	11

A total of 106 to 415 DAPI stained cells successfully transfected with hSIN3B, ETO homologues or AML1-ETO were analyzed. B-23 was used as a marker for nucleoli. A majority of these cells showed a nuclear signal. Nucleoli were visible in 11–20% of the cells (column 4 plus column 5). The ETO homologues showed a nucleolar localization in approximately half of these cells as. AML1-ETO showed no nucleolar localization.

**Figure 8:**
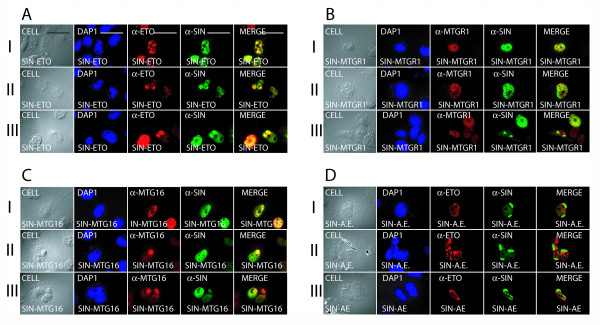
**Colocalization of hSIN3B and ETO homologues**. ETO, MTGR1, MTG16 or AML1-ETO (A.E) was cotransfected with hSIN3B (SIN) in COS-7 cells and analyzed with immunofluorescence microscopy. **(A) **ETO and hSIN3B colocalized in nuclear bodies (I), in the matrix of the nucleolus (II) and at the periphery of the nucleolus (III). **(B) **MTGR1 and hSIN3B were observed separately in nuclear particles and in nuclear bodies respectively (I), but alternatively both were seen at the periphery of nuclear particles (II) or lacked colocalization (III). **(C) **MTG16 and hSIN3B colocalized in nuclear bodies (I), in the matrix of the nucleolus (II) and at the periphery of the nucleolus (III).**(D) **AML1-ETO was observed in the nucleus only whereas hSIN3B was seen in the nucleolus (I – III). Size bars of 10 μM are shown in panel I of part A.

Finally, we examined whether hSIN3B colocalized with the ETO homologues in the nucleolus. Upon coexpression of hSIN3B and ETO, we observed colocalization of both these proteins in nuclear bodies (Fig. 8A, I), the matrix of the nucleolus (Fig. 8A, II) and at the periphery of the nucleolus (Fig. 8A, III). The colocalization between hSIN3B and MTGR1 was not complete (Fig. 7B). Thus, in some cells MTGR1 was present in nuclear particles (Fig. 8B, I) with or without colocalization with hSIN3B at the periphery of these (Fig. 8B, II–III). Complete colocalization was also observed between hSIN3B and MTG16 in nuclear bodies (Fig. 8C, I), in the nucleolar matrix (Fig. 8C, II) and at the periphery of the nucleolus (Fig. 8C, III). No nucleolar colocalization was observed between hSIN3B and AML1-ETO (Fig. 8D) consistent with the lack of interaction between these proteins in IP-Western experiments (Fig. 2D). A quantitative analysis of the data from cotransfection of hSIN3B and ETO homologues is shown in Table 3. A majority of cells showed nuclear colocalization (70–78%). Furthermore, significant colocalization was seen in nuclear bodies, nuclear particles and nucleoli. No colocalization was observed between hSIN3B and AML1-ETO.

**Table 3:** Quantitative analysis of the data on colocalization between hSIN3B and ETO homologues.

Transfection	Cells analyzed	Nuclear colocalization (%)	Nucleolar colocalization (%)	Nuclear body colocalization (%)	Nuclear particle colocalization (%)
SIN – ETO	139	70	14	10	6
SIN – MTGR1	131	77	14	4	5
SIN – MTG16	129	78	18	2	2

A total of 108 to 139 DAPI stained COS-7 cells successfully cotransfected with hSIN3B (SIN) and ETO homologues were analyzed. These cells were divided into % of cells showing nuclear colocalization, nucleolar colocalization, nuclear body colocalization and nuclear particle colocalization.

### Nucleolar localization of hSIN3B and ETO homologues in K562 cells

**Figure 9:**
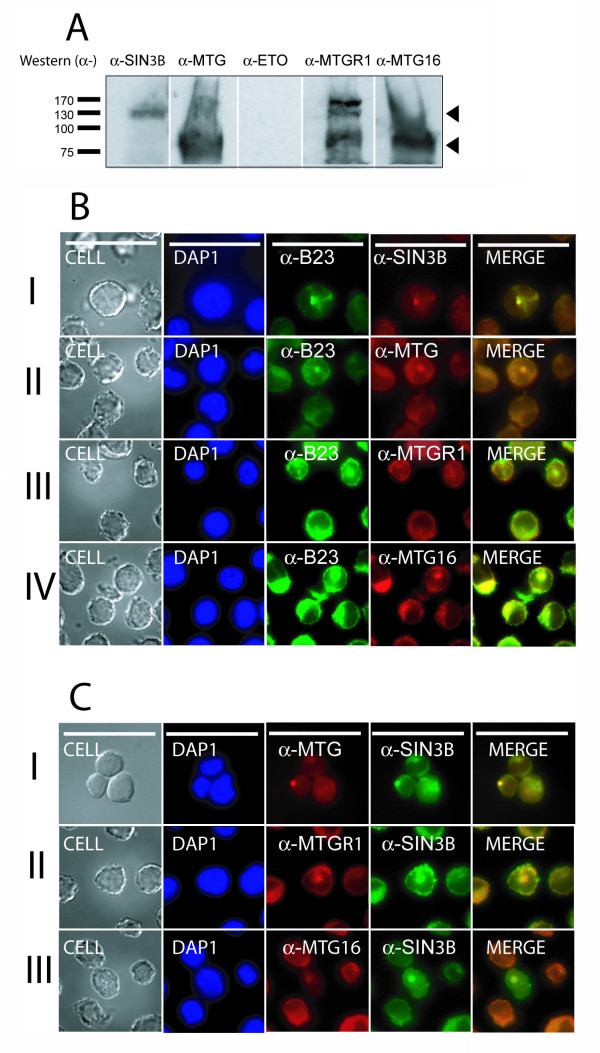
**Nucleolar colocalization of hSIN3B and ETO homologues in K562 cells**. **A**. Expression of hSIN3B (SIN3B) and the ETO homologues in K562 cells as detected by immunoblotting. The α-MTG antibody reacts with all ETO homologues. **B**. Localization of hSIN3B and ETO homologues in the nucleolus. The first column of each panel shows the Nomarski index. The second column shows nuclear staining with DAPI. The third column shows the the B23 nucleolar marker. The fourth column shows nucleolar localization of individual proteins. The fifth column shows a merge of B23 and individual protein. hSIN3B (I) and the ETO homologues (II), MTGR1 (III) and MTG16 (IV) showed colocalization with the B23. **C**. Colocalization of hSIN3B and MTG16 or MTGR1 in the nucleolus. Columns are designed as in B. Size bars of 10 μM are shown in panel I of part B and C.

We confirmed the nucleolar colocalization between hSIN3B and ETO homologues observed upon overexpression in COS-7 cells (Fig. 8) by studies of endogeneous proteins. The HEL human erythroleukemia cell line is the only leukemic cell line that we know of that expresses transcripts for both hSIN3B and all three ETO homologues (this work, [33]), but hSIN3B was not detectable by immunoblotting in these cells (data not shown). Therefore, we used the K562 human erythroleukemia cell line instead although the data will be limited to MTG16 and MTGR1 as this cell line does not express ETO [33]. In support of this, immunoblotting showed the presence of hSIN3B, MTGR1 and MTG16 but not ETO (Fig. 9A). A nucleolar localization of SIN3B, MTGR1 and MTG16 was observed (Fig. 9B), and hSIN3B was shown to colocalize with MTGR1 and MTG16 (Fig. 9C). These observations strengthen our observations that hSIN3B colocalizes with ETO homologues, MTGR1 and MTG16 in the nucleolus.

## Discussion

The major role of SIN3 proteins is to recruit HDACs, which catalyze deacetylation of histones leading to the creation of a repressive chromatin structure [4-7,34]. mSIN3A has been extensively studied as a corepressors, and is known to interact with ETO homologues [11-13,19,20,35,36]. The following observations were made in the present work: (1) The corepressor hSIN3B was shown to be ubiquitously expressed in human tissues and cell lines. (2) Upon ectopic expression, hSIN3B was shown to interact with ETO and MTG16 but not with MTGR1 or AML1-ETO. (3) In primary placenta cells, hSIN3B was found to interact with ETO but not with MTG16 or MTGR1. (4) A nucleolar localization of hSIN3B and ETO homologues was observed both for overexpressed proteins in COS-7 cells and endogenous proteins in the K562 leukemia cell line. Collectively, the results suggest that hSIN3B is a member of a chromatin-repressor complex involving selective ETO homologues.

### SIN3A and SIN3B differ in their interactions with ETO homologues

The region of ETO involved in binding to mSIN3A has been mapped to NHR2 and its flanking regions [11,19]. Our data show that NHR2 is required for an interaction between hSIN3B and ETO. Beyond this, our results also show a role for the amino-terminal part of ETO for an interaction with hSIN3B. This is consistent with the observed lack of an interaction between hSIN3B and AML1-ETO, which is devoid of the 30 amino-terminal residues present in wildtype ETO [37]. However, not only the absence of these residues but also steric hindrance caused by the AML1 part of the chimeric AML1-ETO protein could be important for lack of interaction.

### Interaction between hSIN3B and selective ETO homologues

The corepressor mSIN3A is known to interact with ETO and MTGR1 [11-13,19,20,35]. In contrast to SIN3A, we demonstrated interactions between hSIN3B and ETO or MTG16 but not MTGR1 in COS-7 cells by overexpression. The results of overexpression studies may not necessarily reflect protein interactions as they occur normally. Interestingly, an interaction between hSIN3B and ETO was also detectable in primary cells from the villous part of the placenta. Therefore, our results suggest a non-redundant interaction between hSIN3B and ETO homologues.

### hSIN3B and all the ETO homologues show nucleolar targeting

A nuclear localization of ETO homologues and AML1-ETO has been reported previously [27-29,38-40]. Furthermore, nucleolar targeting of MTG16 but not of ETO and MTGR1 has been reported by Hoogeveen et al. [30]. However, we observed all ETO homologues as well as hSIN3B to be targeted to the nucleolus upon overexpression in COS-7 cells (Fig. 6B). There are intrinsic problems associated with overexpression systems used for these studies wherein the type of plasmid and the efficiency of transfection may influence the results. Importantly, we confirmed the nucleolar colocalization endogenously in the K562 human erytroleukemia cell line.

### Possible role of hSIN3B and ETO homologues in transcriptional inactivation

The periphery of the nucleolar chromatin ring contains a large number of inactive methylated rDNA repeats [41,42]. MTG16 has been demonstrated to be localized at the nucleolar periphery and suggested to play a role in rDNA silencing [30]. In addition, we observed ETO as well as MTGR1 at the nucleolar periphery (Fig. 6, 7). Likewise, we observed a peripheral nucleolar localization of hSIN3B. Furthermore, involvement of the SIN3 corepressor complex in rDNA silencing has been reported [43]. The presence of transcriptional repressors like hSIN3B and ETO homologues in the nucleolus could lead to transcriptional inactivation of rDNA and a slowdown of cell proliferation [44,45].

### Unlike SIN3A, hSIN3B does not interact with AML1-ETO

AML1-ETO is known to suppress AML1-responsive gene transcription [46]. AML1-ETO has been shown to interact with mSIN3A [12,13]**, **but our data show that it does not interact with hSIN3B. This seems to be explained by the deletion of the amino-terminus of ETO in AML1-ETO as an aminoterminal deletion of 30 aminoacids abrogated the interaction between ETO and hSIN3B. Previous [38-40] and present studies show lack of targeting of AML1-ETO to the nucleolus. This is in contrast to the nucleolar targeting of ETO. Furthermore, upon coexpression with hSIN3B, AML1-ETO and ETO showed separate nuclear localization (Fig. 7A and 7D). Therefore our data suggest that AML1-ETO is not a part of a possible hSIN3B-associated complex.

## Conclusion

Taken together, our data indicate that hSIN3B is a potential member of a core repressor complex involving the ETO homologues. The NHR2 domain of ETO and its flanking regions are involved in making contact with the corepressor mSIN3A [11,19] and our data suggests that NHR2 and the amino-terminus of ETO are required for contact with hSIN3B. This difference may be attributed to the difference in the structure of the PAH2 domain of SIN3 homologues [47]. We speculate that a corepressor complex involving ETO homologues may contain either SIN3A or SIN3B. It is unlikely that both SIN3A and SIN3B are part of the same complex involving ETO homologues. Previous studies have shown that ETO pulls down a complex of about 600 kDa that contains mSIN3A, NCoR and HDACs [12]. A nucleolar repressor complex of this limited size is unlikely to be able to hold both SIN3A and SIN3B, as both are large proteins. We propose a model in which a corepressor complex contains either hSIN3A or hSIN3B, but not both. The ETO homologues of this complex may be interchangeable. Finally, the finding of hSIN3B as an interacting partner for specific ETO homologues in the nucleolus suggests an epigenetic control of nucleolar transcriptional regulation.

## Methods

### Reverse transcription PCR

The expression of hSIN3B in various human tissues and cell lines was determined by reverse transcription PCR (RT-PCR). Human fetal and adult multiple-tissue cDNA (MTC) panels (Clontech Laboratories Inc., Palo Alto, CA, USA) and cDNA prepared from various hematopoetic cell lines were used as template. For preparing cDNA from cell lines, total RNA was extracted from cells using the RNeasy mini kit (Qiagen, Hilden, Germany). The Omniscript RT kit (Qiagen, Hilden, Germany) was used for synthesis of cDNA from one microgram of RNA using random hexamer as primer, following the manufacturers' instructions.

PCR parameters were: 32 × (95°C for 30 s, 68°C for 2 min) followed by extension at 68°C for 3 min. The chosen primer sequences unique to hSIN3B were situated on separate sides of an intron, in order to remove the possible amplification of genomic DNA. The sequences of the forward and reverse primer were – 5'-GCCACGAGAAGCTGCCGGTGCACGTA-3' and 5'-CACCGTCCCCGTGGTTGTGCGAATTCT-3'. Amplification of G3PDH was used as internal control for all the tissues. All PCR reactions were done in triplicate from two independent MTC panels.

### Real-time RT-PCR

For real-time RT-PCR of hSIN3B and ETO homologues, human fetal and adult multiple-tissue cDNA (MTC) panels were used as templates. Taqman Universal PCR Master mix and 7,000-sequence detection system were used (Applied Biosystems, CA, USA). Primer and probe concentrations were 0.9 μM and 0.25 μM, respectively. PCR parameters were: 50°C for 2 min, 95°C for 10 min and 40 × (95°C for 15 s and 60°C for 1 min). For hSIN3B, primer and probe mix were from Applied Biosystems Assay on demand™ Hs00391562_m1. The real-time RT-PCR for the ETO homologues was carried out as previously described [33].

The threshold cycle (C_t_) was determined for all samples. A standard curve was generated by linear regression of the C_t _values from cDNA of the HEL cell line; the corresponding RNA amounts ranged from 1 pg to 100 ng. The 1 pg standard was the denominator, set to 1. Based on the C_t _values of the samples, transcript levels were derived from the standard curve and normalized against the normalizing standard G3PDH. For statistical comparison between tissues, the transcript level of fetal kidney was kept as 1. The PCR reactions were done three times, each one in triplicate from MTC panels. Standard deviation was calculated from the mean of three experiments and plotted as error bars.

### Plasmids

The coding sequence of hSIN3B was amplified from cDNA in clone fh15187 (KIAA0700 in pBluescript from Kazusa DNA Research Institute, Japan). Restriction cleavage sites were introduced at both ends of the coding sequence. The PCR products were digested with Mlu1 and SalI, and cloned into the pCMV5 vector. The coding sequences of MTGR1a and MTG16a (kind gifts from Dr. F. Morohoshi) were introduced into pCMV5 as previously described [33]. pCMV5_ETOb and pCMV5 vectors with ETO NHR1-4 deletion mutants (pCMV5_ETOΔTAF, pCMV5_ETOΔHHR, pCMV5_ETOΔNervy and pCMV5_ETOΔMYND) were kind gifts from Dr. Scott Hiebert. The coding sequences of ETO, MTGR1 and MTG16 were also introduced after PCR amplification with V5 tags into pCMV5 vector (pCMV5_ETO-V5, pCMV5_MTGR1-V5 and pCMV5_MTG16-V5). A new deletion mutant of pCMV_ETO lacking the first 30 aas was also constructed after PCR amplification (pCMV_ETO-30). All the constructs were sequenced using Big Dye Terminator cycle sequencing kit (version 3.1) (Applied Biosystems, CA, USA).

### Cells and transfection

HEL, K562, U937, NB4, THP and HL60 cells were grown in a 5% CO_2 _atmosphere at 37°C in RPMI – 10% FBS and COS-7 cells were grown in DMEM -10% FBS (Gibco BRL, MD, USA). Cell counting was done in a Burker chamber and viability was determined by Trypan blue exclusion. COS-7 cells were transfected using Polyfect (Qiagen, Hilden, Germany) according to the manufacturer's instructions. For Western blotting and IP-Western 2.5 × 10^6 ^cells were seeded in a 10 cm^2 ^Petri dish, transfected the next day with 10 μg of plasmid and analysed 48 h later. For immunofluorescence staining, 0.4 × 10^6 ^COS-7 cells were seeded in a six-well plate, transfected the next day with 1.2 μg of plasmid and allowed to grow for another 24 h before analysis. For immunofluorescence staining on endogenous proteins in K562 cells, 1 × 10^6 ^cells per well were fixed in 96-well plate.

### Collection of placental tissue and nuclear extraction

Placental tissue was collected at the Department of Obstretics and Gynecology, Lund University hospital. Sampling was performed after informed consent as approved by the Ethical Committee review Board for studies of human subjects. Tissue was taken from the central villous part of the placenta as described [48]. For nuclear extraction (all steps at 4°C), 150 mg of fresh placental cells were homogenized 3 times for 10 sec each, with intermittent cooling in 3 ml of a cell lysis buffer [10 mM HEPES-KOH (pH 7.9), 1.5 mM MgCl_2_, 10 mM KCl, 0.1% NP-40] supplemented with 0.5 mM DTT and 0.2 mM PMFS. The homogenate was left on ice for 1 h and centrifuged at 3,400 × g for 4 min. The cell pellet was extracted with 1.5 ml of a nuclear lysis buffer [20 mM HEPES-KOH (pH 7.9), 1.5 mM MgCl_2_, 0.2 mM EDTA, 420 mM NaCl, 25% Glycerol] supplemented with 0.5 mM DTT and 0.2 mM PMFS. The lysate was kept on ice for 1 h and centrifuged at 10,000 × g for 15 min. The nuclear extract thus obtained was diluted 1:20 in a lysis buffer [250 mM NaCl, 20 mM Na-phosphate (pH 7.0), 30 mM Na-pyrophosphate (pH 7.0), 5 mM EDTA, 0.1 mM Na_3_VO_4_, 10 mM NaF, 0.1% NP-40].

### IP-Western blotting

Cells were lysed in 250 mM NaCl, 20 mM Na-phosphate, 30 mM Na-pyrophosphate (pH 7.0), 5 mM EDTA, 0.1 mM Na_3_VO_4_, 10 mM NaF, 0.1% NP-40 – supplemented with protease inhibitors (lysis buffer) (Roche, Germany). The lysate was incubated on ice for 1 h, cleared by centrifugation, and incubated overnight with 1 μg of α-hSIN3B, α-ETO, α-MTGR1, α-MTG16 or α-MTG, and proteinA-Sepharose (Amersham Pharmacia, Sweden). Immunoprecipitates were subjected to SDS-polyacrylamide gel electrophoresis (PAGE) on a precast 10–20% Tris-Glycine gel (Novex, CA, USA) and transferred to a PVDF membrane (Amersham Pharmacia, UK). The filters were incubated with α-hSIN3B (SC-13145, Santa Cruz Biotechnology Inc., USA; dilution 1:800), α-ETO, α-MTGR1, α-MTG16 or α-MTG (dilution 1:1000) and incubated with horseradish peroxidase-conjugated secondary antibody (Bio-Rad, CA, USA). Rabbit pre-immune sera (PIS), rabbit polyclonal antibodies to ETO homologues (α-ETO, α-MTGR1 and α-MTG16) and the rabbit polyclonal antibody α-MTG recognizing all three ETO homologues were produced as previously described [26].

For Western blotting of placental proteins, the tissue was homogenized in TRIZOL (Invitrogen, UK). The manufacturer's instructions were followed for extracting the protein. After a final wash the protein pellets were dissolved in a buffer containing 2% CHAPS and 8 M Urea for 2 h. The protein concentrations were measured using BCA protein assay kit (Pierce, IL, USA). Blots were treated with recycling kit (Chemicon, CA, USA) in order to reprobe with α-actin (Santa Cruz Biotechnology Inc., CA, USA). Western blots were developed using an ECL kit (Amersham Pharmacia, UK).

### Immunofluorescence microscopy

24 h after transfection, 0.2 × 10^6 ^COS-7 cells were redistributed in a 12-well plate containing coverslips. The following day, the cells were washed twice with 1 mL cold PBS, fixed with 400 μL 2% [wt/vol] paraformaldehyde solution and incubated 30 min at room temperature. After fixation, the cells were permeabilized in 400 μL 0.5% [vol/vol] Triton X-100 in PBS for 1 h at room temperature and thereafter incubated in 300 μL blocking solution (PBS containing 0.1% bovine serum albumin [wt/vol], 0.2% Tween 20 [vol/vol] and 5% goat serum [vol/vol]) for 1 h at room temperature. Next, the cells were incubated at room temperature for 1 h with the primary antibodies – α-hSIN3B, α-ETO, α-MTGR1, α-MTG16 (dilution 1:1,200) or goat primary anti-B23 (dilution 1:250; Santa Cruz Biotechnology Inc., CA, USA) – in blocking solution. Following washing, cells were incubated for 1 h with secondary antibodies – goat anti-rabbit Alexa Fluor 488; goat anti-mouse Alexa Fluor 488, goat anti-mouse Alexa Fluor 594, donkey anti-goat Alexa Fluor 594 (dilution 1:1,500, Eugene, OR, USA).

K562 cells were stained in suspension and attached to poly-L-lysine-coated coverslips after staining. Briefly, 1 × 10^6 ^cells were washed with Na-medium (5.6 mM glucose, 127 mM NaCl, 10.8 mM KCl, 2.4 mM KH2PO4, 1.6 mM MgSO4, 10 mM HEPES, and 1.8 mM CaCl2, pH adjusted to 7.3 with NaOH). The cells were then fixed using 1% paraformaldehyde solution. The cells were incubated in blocking buffer 1 (1% BSA (w/v) and 5% (v/v) goat serum in Na-medium) for 1 h. Subsequent incubation was done in blocking buffer 2 (Na-medium containing 1% BSA (w/v), 0.02% Triton X-100, 0.2% Tween 20 (v/v), and 5% (v/v) goat serum) for 30 min at room temperature. Next, cells were treated with the primary antibodies in blocking buffer 2 in conditions described above. Following washing, cells were incubated with the secondary antibodies for 1 h.

After washing, cells were overlaid with ProLong Antifade reagent with 4'-,6-Diamidino-2 Phenylindole (DAPI, Eugene, OR, USA) to counterstain nuclei before mounting. Images were recorded on a Nikon Eclipse TE300 inverted-fluorescence microscope (Nikon, Tokyo, Japan) equipped with a Hamamatsu C4742-95 cooled CCD camera, using a Plan Apochromat 100× objective and a high-numerical-aperture oil condenser. Adobe Photoshop (Adobe Corp., San Jose, CA, USA) was used to pseudocolor and overlay the recorded images.

## Authors' contributions

RSD initiated the project, designed & performed experiments, analyzed data and prepared the manuscript. SRL designed and performed experiments. IO was involved in experimental design and manuscript preparation. All authors read and approved the final manuscript.
